# Editorial

**Published:** 1864-07

**Authors:** 


### Editorial.

### FILLING PULP CAVITIES AND CANALS.

There is a great diversity of opinion in regard to the proper time to fill teeth, the pulps of which have been destroyed; hence there are many phases of practice. This diversity is; owing, in a great measure, to the fact that there is no definite or even well-settled conviction on the subject. A clear knowledge on this subject is attainable, and very important, and should be possessed by every one who assumes to perform such operations as are here contemplated.

A diversity in practice shows a difference of opinion, and this is evidence that some body's knowledge is deficient or incorrect. There can not be a great diversity of opinion, and yet all based upon a knowledge of truth.

Now the question occurs: What is the proper time to fill the pulp chambers and canals of teeth, where the operation is required? And we are often asked: "How soon do you fill teeth after destroying the nerves?" There is no rule or method of proceedure that will apply to all cases, or that may be adopted for general practice.

The only correct practice can proceed from a thorough knowledge of the subject—a familiarity with and a clear understanding of physiological and pathological conditions, together with the extent and power of modifying influences. It is impossible to give an answer to these queries without specifying cases.

With a healthy subject, of good vital force, if the pulp is destroyed by an operation—and that is the better method, especially in the six interior teeth, and very often in others—fill the tooth at the same sitting in which the pulp is removed.

If the hemmorrhage does not cease soon after the excision of the pulp, arrest it with the use of any good hæmostatic; then introduce a few fibres of flax, asbestos or cotton, slightly moistened with creasote. Let this be forced into the fang till there is no cavity beyond it, then fill.

We only delay filling after the operation of removing a nerve, in those cases that are very susceptible to irritation and inflammation. They, however, are of not very frequent occurrence, and when such a condition does exist, it is better to anticipate the difficulty by proper treatment.

In cases where the pulp is destroyed by arsenious acid, or any agent of that kind, the tooth should be filled immediately after the removal of the pulp. We think nothing is gained by delay, except, perhaps, in those cases that are very impressible under the exhibition of arsenic, and in these cases it is only requisite to delay because the irritation resulting from manipulation might increase the difficulty which had its beginning with the arsenic. Perhaps it is better, in such cases, to refrain from filling till the arseincal irritation has subsided. Arsenious acid, if used judiciously for devitalizing pulps of teeth, will seldom produce soreness.

In those cases where the pulp is destroyed, previous to the dentist's knowledge of the case, the greater difficulty is found. In these cases, when the health of the patient is good, and no abscess from the tooth, and no sloughing or discharge of pus through the fang, the tooth may be prepared and filled throughout at once. If there is an abscess from the tooth, it may be filled, in many instances, at once, and the abscess treated through the discharging channel. If, however, there is a discharge through the fangs of a tooth, whether there is an abscess or not, it must receive such treatment as will arrest that discharge before being filled. The length of time required to accomplish this will be very various, dependent upon the mode of treatment, and the health and the constitutional peculiarities of the patient. In all cases the arrest of the discharge should be complete before filling.

It is true, that in some very favorable cases, the absorbents would be able to take up, and convey away, some slight remaining discharge; yet, in the great majority of cases, such slight discharges, denied an egress, would result in inflammation and alveolar abscess, so that it is the best practice, in all cases, to have the discharge completely arrested, and all the parts as nearly healthy as possible before filling. T.

### THE ANNUAL MEETINGS.

Our readers, we hope, will bear in mind, the meeting of the American Dental Association which takes place on the last Tuesday of this month (July) at Niagara Falls. We anticipate a large gathering of the profession—of its workers.

There is a large amount of work to be accomplished at that meeting, and we doubt not it will be of great interest to the profession. The indications are that there will be a much larger meeting of this body than ever before. Within the last year there have been several new societies formed, all of which have appointed delegates. We think there has been more care in selecting delegates by all the societies this year than ever before—selecting only those who will make it a point to go.

Let each and every one go resolved and prepared to do something.

The American Dental Convention meets on the first Tuesday of August at Detroit, when we also anticipate a very large meeting. Here there is a large opportunity for a spontaneous outburst of professional enthusiasm. We value these meetings very much for the opportunity afforded for cultivating the social element. The trip from Niagara to Detroit can be made one of the features of the occasion, as we doubt not it will. No doubt the profession of the West will be out in its strength. The following subjects are those proposed for discussion at the Convention, viz:

1. The best means of improving the practice and elevating the profession of dentistry; 2. Anæsthetics—their proper use and relative value; 3. Extracting teeth: when it should be done and when not,—the best instruments for the purpose and the subsequent treatment, when any is required; 4. Absorption of alveolar process—causes and treatment; 5. Filling teeth; the relative value of different materials, and the mode of operating in difficult cases; 6. The best method of obtaining accurate impressions and models of the mouth; 7. The relative value of different materials as a base for artificial teeth; 8. Miscellaneous. T.

### GOLD FOIL.

We have for some time been using gold foil manufactured by J. B. Dunlevy of Pittsburg, and find it to be a most excellent article, inferior to none we have ever used. He makes three qualities, non-adhesive, medium adhesive, and very adhesive. We have used each of these, and each is very good of its kind. We have used several ounces and find it entirely uniform, which is a great desideratum.

Mr. D. gives his personal supervision to every manipulation and process in the manufacture of his foil, so that every sheet has his mark upon it.

We have used more of the very adhesive, it welds most readily, and very perfectly. In speaking thus, we do not derogate from any other foil, for we know there are many very good foil makers. Our nature is such that when we find a very good thing we must speak right out about it. T.

### INSTRUMENTS.

Always when we get a good instrument, either plugger or excavator, that works finely and just to our notion, we wish every professional brother in the country had one just like it; but as we can't furnish every one thus, we'll do the next best thing we can, and that is, "tell about it." We, a few days ago, received from S. S. White some serrated pluggers, that of their kind we have never seen equaled; they fill a niche or two, that hitherto, with us, at least, have been vacant; some of them work in, where we have had nothing to enter before. They are smooth and beautifully cut, and consolidate gold admirably, without dragging; they have no tricks except to do the thing right.

About the same time we also received some of his diamond point excavators, which are "magnificent." We hope everybody will try these instruments; they will then learn a good deal more than we have ventured to say. T

